# Psychosocial stressors and protective factors for major depression in youth: evidence from a case–control study

**DOI:** 10.1186/s13034-020-0312-1

**Published:** 2020-02-08

**Authors:** Charlotte Elisabeth Piechaczek, Verena Pehl, Lisa Feldmann, Stefan Haberstroh, Antje-Kathrin Allgaier, Franz Joseph Freisleder, Gerd Schulte-Körne, Ellen Greimel

**Affiliations:** 1 Department of Child and Adolescent Psychiatry, Psychosomatics and Psychotherapy, University Hospital, LMU Munich, Waltherstr. 23, 80337 Munich, Germany; 2 Department of Child and Adolescent Psychiatry, Psychosomatics and Psychotherapy, University Hospital, LMU Munich, Pettenkoferstr. 8A, 80336 Munich, Germany; 3 Department of Human Sciences, Institute of Psychology, Bundeswehr University Munich, Werner-Heisenberg-Weg 39, 85577 Neubiberg, Germany; 4 Department of Child and Adolescent Psychiatry, Psychosomatics and Psychotherapy, University Hospital, LMU Munich, Nußbaumstr. 5A, 80336 Munich, Germany; 5 KBO Heckscher-Klinikum, Deisenhofenerstr. 28, 81539 Munich, Germany

**Keywords:** Psychosocial, Stressors, Protective factors, Major depression, Youth

## Abstract

**Background:**

Severe adverse life events, such as traumatic experiences, are well-known stressors implicated in (youth) major depression (MD). However, to date, far less is known about the role of more common psychosocial stressors in the context of MD, which are part of everyday life during youth. In addition, it is not well-understood whether and how distinct stressors interact with protective factors in youths diagnosed with MD. Thus, the present study aimed at examining several specific psychosocial stressors implicated in a first-episode juvenile MD and addressed the question whether protective factors might moderate the relationship between stressors and a diagnosis of MD.

**Methods:**

One-hundred male and female youths with MD and 101 typically developing (TD) controls (10–18 years) were included. A large number of qualitatively different psychosocial stressors occurring in various areas of life were assessed via self-report. Moreover, we also investigated sociodemographic and pre- and postnatal stressors, as well as the presence of familial affective disorders via parental-report. Social support and a positive family climate were conceptualized as protective factors and were assessed via self-report.

**Results:**

Results showed that the proportion of youths experiencing specific psychosocial stressors was higher in the MD than in the TD group. In particular, the proportion of youths indicating changes at home or at school, experiences of violence, delinquent behavior, as well as the proportion of youths who were exposed to sociodemographic stressors was higher in the MD than in the TD group. Moreover, the percentage of youths with a family history of an affective disorder, or whose mothers experienced psychological burdens during/after pregnancy was elevated in the MD group. Youths with MD experienced less social support and a less positive family climate than their TD peers. These factors, however, did not buffer the influence of specific stressors on MD.

**Conclusion:**

We could show that next to more severe adverse life events, more common psychosocial stressors are linked to youth MD. Importantly, by identifying distinct stressors in youth MD, our results can increase treatment and prevention efforts aiming to improve the outcomes in youths affected by MD or in at-risk individuals.

## Background

Major depression (MD) is one of the most common and debilitating psychiatric disorders worldwide [1]. The onset can often be traced back to adolescence with prevalence rates of about 8% [2, 3]. Suffering from MD early in life often seriously affects later development, as evidenced by e.g., school dropout and lower life satisfaction [4, 5].

Besides genetic and other (e.g., cognitive) factors, psychosocial stressors are known to play an important role in the etiology of the disorder [6, 7]. Due to the pervasiveness, impairments and high prevalence of youth MD, it is important to identify specific psychosocial stressors related to the disorder during this developmental period. Insight into such factors might increase treatment and prevention efforts. To date, knowledge concerning the relationship between specific stressors and depression predominantly originates from studies in (young) adults with MD or from non-clinical youth samples with elevated depressive symptoms. However, it remains unclear to what extent these results can be generalized to youths with a diagnosis of MD.

Previous studies on psychosocial stressors implicated in MD mainly focused on stressful life events (SLEs), defined as “cluster of social events requiring change in ongoing life adjustment” [8]. SLEs, such as the death of a loved one or a serious illness, are supposed to play a causal role in the onset of juvenile MD [7, 9].

With regard to SLEs in the context of family life, there are conflicting results concerning the predictive value of parental separation, divorce or living in a one-parent family for MD and depressive symptoms during youth or young adulthood [10–16]. Related to this, findings are also inconclusive with regard to the role of experiences of loss (primarily in respect to the death of a parent) in the development of depressive symptoms and MD in youths and adults [14, 17–19].

Investigations on school-related SLEs as predictors for MD in youth are relatively scarce. Prior findings on the predictive value of specific school-related events (e.g., repeating a grade or having to change school) for MD in youth are mixed [16, 18]. Previous studies indicate that distinct stressful events due to low academic achievement (e.g., repeating a grade) predict MD in young adulthood [19]. In this context, it needs to be emphasized that the effects of low academic achievement on the risk of MD are mainly evident in girls and show a bidirectional relationship with depressive symptoms [19–21].

With respect to experiences of violence, there is robust evidence showing that the exposure to sexual or physical abuse are predictors of MD and depressive symptoms in youth [22–24]. The effects of violence on youth depression have been shown to be enduring. In line with this, evidence suggests that elevated depressive symptoms and episodes of MD may even persist up to two years after having experienced incidences of violence [24, 25].

Besides SLEs, other psychosocial factors may also play a role in youth MD. These factors encompass delinquent behavior, familial psychopathology, and birth-related, as well as sociodemographic factors. Results concerning the role of delinquent behavior in depressive symptoms in youths are inconclusive. Studies have identified delinquent behavior as an antecedent of depressive symptoms in male youths and young adult males. However, this finding does not seem to apply for females [13, 26]. Moreover, evidence suggests that the relationship between delinquent behavior and depressive symptoms is rather bidirectional, with depressive symptoms resulting in delinquent acts and vice versa [26, 27].

Studies investigating psychosocial birth-related aspects have identified emotional distress of the mother during pregnancy as a risk factor for juvenile MD [28–30]. However, this result has not always been confirmed [31]. In addition, the occurrence of a maternal postpartum depression has also been identified as a highly relevant factor contributing to MD and internalizing problems in juvenile offspring [32–34]. However, there is also evidence showing that the relationship between a maternal postpartum depression and MD in youth is substantially mediated by a later maternal MD [32]. Related to this issue, there is a large body of literature showing that parental depression is a major risk factor for MD in youth offspring [35–38]. Intergenerational transmission of depression might be due to multiple mechanisms, such as neurobiological, behavioral, cognitive, and genetic pathways [37, 39].

Regarding sociodemographic stressors, a low parental socioeconomic status does not seem to be a factor contributing to depressive psychopathology in youth and MD in young adults [19, 40]. However, specific factors constituting the socioeconomic status have in part been found to predict youth MD. In particular, low parental education has been reported to be a risk factor for depressive symptoms and MD in youth [12, 41], but this has not always been found [16]. Moreover, parental unemployment is implicated in depressive symptoms and youth MD [12, 42]. Results concerning the predictive value of migrant status of the parents on depressive symptoms and MD in youth are mixed [16, 42].

Discrepant findings in studies investigating psychosocial stressors associated with youth MD may be explained by different factors including, e.g., (1) differences in age (youth vs. adulthood), (2) differences in defining and assessing psychosocial stressors, as well as (3) the definition of depression (MD vs. depressive symptoms).

In addition to investigating psychosocial stressors, a number of prior studies examined factors that may protect youths from developing MD. Among other factors, research in this domain has focused on social support and family climate. A positive family climate and social support have been supposed to act as protective factors in relation to overall psychopathology, and in particular regarding depressive symptoms and MD in youth [43–45]. In line with this notion, it is also generally assumed that social support may attenuate the effects of psychosocial stressors on depressive symptoms [46]. However, most empirical studies failed to find a buffering effect of social support and a positive family climate [47–49]. These findings indicate that psychosocial stressors and social support/a positive family climate seem to independently influence the risk of depressive symptoms and MD in youth (but see [50] for contradicting findings). However, most prior studies were restricted to non-clinical youth samples with elevated depressive symptoms [47–49]. Thus, it remains unanswered whether these findings can be transferred to youths with a clinical diagnosis of MD. The only prior study that investigated the buffering effect of social support in clinically depressed youths and that was based on a prospective design comprised a relatively small sample (*N* = 24) [47]. To date, it remains an open question whether the buffering effect can be found in a larger sample of clinically depressed youth.

As summarized above, most results on psychosocial stressors and their interplay with protective factors originate from adult MD samples or from samples of youth with elevated depressive symptoms. However, results from studies investigating youths with heightened depressive symptoms cannot be transferred to youths with MD [51]. Similarly, psychosocial stressors implicated in MD during adulthood may not be congruent with psychosocial stressors for youth MD [52]. In this context, it needs to be emphasized that youth is characterized by changes in biological systems (e.g., the maturation of stress systems), as well as an increase of psychosocial stressors [53, 54]. Therefore, this phase is considered an especially sensitive developmental period conveying a heightened risk of psychiatric disorders, including MD. Thus, it seems important to gain a deeper insight into psychosocial stressors and protective factors implicated in youth MD based on a well-characterized clinical sample.

Accordingly, the first aim of this study was to investigate specific psychosocial stressors in youth with MD and to identify the most relevant stressors for this patient group. The second aim was to examine whether social support and a positive family climate act as protective factors in youth MD and to investigate whether these factors moderate the relationship between specific psychosocial stressors and MD.

Building on prior findings, we hypothesized that the proportion of youths who experienced psychosocial stressors would be higher in the MD compared with the TD group. Specifically, we expected that the portion of youths experiencing violence would be higher in the MD than in the TD group [24]. Additionally, we hypothesized that affective psychopathology would be increased in families of youths with MD, as compared with TD youths [28, 30, 36]. Finally, we hypothesized that sociodemographic stressors would be more prevalent in the MD group [12, 41, 42]. We also expected that TD youths would experience more social protective factors than youths with MD [44, 45]. We did not state a directed hypothesis regarding the buffering effect of these protective factors on the link between psychosocial stressors and MD due to the scarce and mixed previous findings [47–50].

## Methods

### Participants

The study sample forms part of a larger study on the genetic bases of unipolar depression in children and adolescents. One-hundred youths with a current first-onset MD and 101 age- and sex-matched TD controls aged 10–18 years were included in the present study. Table 1 depicts the age- and sex distribution in the current sample.

**Table 1:** Demographic and clinical characteristics of the study sample

	Youths with MD (*n* = 100)	TD youth (*n* = 101)	*p*
Age (*M*, *SD*)	15.21 (*1.65*)	15.52 (*2.15*)	.261
Age range	10–17	10–18	
Sex (m/f)	24/76	25/76	.901
Depressive symptoms^a^			
BDI-II sum score (*M*, *SD*)	29.67 (*13.77*)	n.a.	
DIKJ T-value (*M*, *SD*)	68.00 (*10.54*)	n.a.	

Sociodemographic factors were conceptualized as stressors and are depicted in Table 2

*MD* major depression, *TD* typically developing, *M* Mean, *SD* Standard deviation, *BDI*-*II* Beck Depression Inventory II, *DIKJ* Depression Inventory for Children and Adolescents

^a^Youths ≤ 12 years filled in the DIKJ, youths > 12 years filled in the BDI-II

The MD group was recruited from two child and adolescent psychiatry clinics. Inclusion criteria were sufficient German language skills, intellectual capacity to complete the questionnaires, and a diagnosis of a current first-onset MD, which was assessed by a standardized diagnostic interview (see “Measures” section). According to ICD-10, 18 subjects had a mild depression, 26 a moderate depression and 56 a severe depression.

Patients with a current or past attention deficit/hyperactivity disorder (ADHD), schizophrenic disorder, bipolar disorder or a pervasive developmental disorder were excluded. MD patients with other comorbid diagnoses than the above listed were included if MD was the primary diagnosis. The frequencies of current and past comorbid diagnoses are included in the Additional file 1.

The TD group was recruited via address lists of former study participants and the hospitals’ websites. The inclusion criteria were sufficient German language skills, intellectual capacity to complete questionnaires, and no past or current mental illnesses. Mental disorders were excluded based on the same standardized diagnostic interview as applied in the MD group (see “Measures” section).

The participants obtained a 20 Euro voucher as a compensation for their effort. The study was approved by the ethics committee of the Medical Faculty of the University Hospital Munich. The study was in accordance with the guidelines laid down in the Declaration of Helsinki and in compliance with national legislation. All participants were informed in detail about the design and the aims of the study, and gave written assent to participate. Written informed consent was also obtained from at least one parent/legal custodian, after the parent(s)/legal custodian(s) had been informed about all aspects of the study.

### Measures

#### Diagnostic interview

Diagnoses of MD and potential comorbid psychiatric disorders based on ICD-10 [55] were made using a standardized semi-structured interview (Kinder-DIPS; [56]), that was administered to the youth and to one parent. The Kinder-DIPS is a well-established German diagnostic interview with previous data showing high test–retest-reliabilities (Cohen’s κ = .85−.94 for the parent-version and Cohen’s κ = .48−.94 for the youth-version for all psychiatric diagnoses; [57]). Interviewers were psychologists who had earned an official certificate after having completed a Kinder-DIPS training.

#### Dimensional assessment of depressive symptoms

To assess the severity of the depressive episode, youths with MD between 10 and 12 years (*n* = 14) completed the Depression Inventory for Children and Adolescents (DIKJ; German version: [58]), while youths older than 12 years (*n* = 84) filled in the Beck Depression Inventory—second edition (BDI-II; German version: [59]). The DIKJ and the BDI-II are established measures of depression symptom severity with good internal consistency (Cronbach’s α = 0.84 [58] and 0.93 [59], respectively). As can be expected, youths with MD scored higher on the DIKJ/BDI-II compared with TD youths (see Table 1).

#### Questionnaire on psychosocial stressors

A comprehensive questionnaire was administered to both the participants (self-report questionnaire) and to one of the parents (parental-report questionnaire) to assess psychosocial stressors. The questionnaire was adapted from the Life Event Survey [60] and the Munich Event List (MEL; test–retest reliability: κ = 0.85; [61, 62]). Face and content validity are assumed as we assessed stressors, which are common and relevant during youth [63]. As with most questionnaires assessing life events, calculating the internal consistency would not be appropriate [64, 65].

In the self-report questionnaire, psychosocial stressors concerning changes at home or at school, death of a loved one, experiences of violence and delinquent behavior were assessed, whereby questions were asked in past terms (for items, see Table 2). Parents answered questions on psychosocial burdens during/after pregnancy, affective disorders in the family as well as on sociodemographic stressors (for items, see Table 2). 78.6% of the parental-report questionnaires were answered by the mothers. The answer format of each of the above-mentioned items was coded dichotomously (“yes”/”no”).

#### Questionnaires on protective factors

To assess protective factors, two questionnaires on social support and family climate were administered to the participants. The social support questionnaire was adapted from the MOS Social Support Survey [66]. For reasons of brevity, this questionnaire contained 10 items of the original 20 items to measure social support (e.g., “Is there someone who loves you and who gives you the feeling of being loved and needed.”). For each item, participants were asked to indicate how often social support was available to them. Response options were: “none of the time”, “a little of the time”, “some of the time”, “most of the time”, and “all of the time”. The original questionnaire has very good reliability (Cronbach’s α = .95; [66]).

The questionnaire administered to assess family climate was taken from the Children’s Health Survey in Germany and was based on the Family Climate Scales (KiGGS; [67, 68]). Youths in both groups had to answer 21 questions about family climate; for example, “In our family everyone has the feeling that one is listening to him and pays attention to him”. Response choices were: “none of the time”, “a little of the time”, “some of the time”, “most of the time”, and “all of the time”. The family climate scale has been reported to show acceptable reliability (Cronbach’s α = .76; [67]).

To investigate the factor structure of the two composite scales “Social support” and “Family climate” in the present sample, two separate exploratory factor analyses were conducted (for a detailed description and results, see Additional file 2). We also calculated the internal consistency of the scales that were revealed in the factor analyses. Results from these calculations can also be found in the Additional file 2.

### Data analysis

SPSS for Windows was used to conduct statistical analyses. The first aim of the study was to identify psychosocial stressors implicated in youth MD and to subsequently establish the most relevant stressors. Due to the substantial number of stressors included in the present investigation, we defined several psychosocial stress domains under which the specific psychosocial stressors were grouped. A detailed description of the grouping approach is summarized in the Additional file 3. Table 2 lists the stressors investigated and their assignment to the stress domains.

In a next step, we tested differences between the MD and the TD group regarding the proportion of individuals who were exposed to the respective stress domain (i.e., the proportion of individuals who experienced at least one psychosocial stressor within the stress domain) using *χ*^2^-tests.

Since we aimed to investigate group differences (MD vs. TD group) with regard to *specific* psychosocial stressors, we then conducted follow-up *χ*^*2*^-tests in case the *χ*^*2*^-test for the respective stress domain yielded a significant result. To correct for multiple testing, the Bonferroni-Holm correction was applied both at the level of the global stress domains as well as at the level of individual stressors within the respective domains.

Following our first study aim, we focused on the identification of the most relevant psychosocial stressors for our youth MD sample. Therefore, we conducted a binary logistic regression analysis with group (MD/TD) as dependent variable and specific psychosocial stressors used as independent variables. This analysis was restricted to individual stressors for which significant group results emerged in the *χ*^*2*^-tests.

Our second study aim was to examine whether social support and a positive family climate act as protective factors in youth MD and whether these factors moderate the relationship between specific psychosocial stressors and case–control status. To accomplish this, we first examined group differences in protective factors. We therefore ran a multivariate analysis of variance (MANOVA) with the sum scores of the scales “Positive Family Climate”, “Activities”, and “Control” as dependent variables and group (MD/TD) as between-subjects factor. In the case of a significant group effect in the MANOVA, follow-up univariate analyses of variance (ANOVAs) were conducted, thereby applying the Bonferroni-Holm correction to correct for multiple testing. To examine group differences in social support, the sum scores of the “Social Support” scale were compared between groups using an independent samples *t*-test.

To investigate potential buffering effects of the scales “Social Support” and “Positive Family Climate” on the relationship between specific psychosocial stressors and group (MD/TD), moderation analyses were conducted using Hayes’ Process macro for SPSS (Model 1; [69]). Moderation analyses were restricted to the stressors that were found to be significant in the regression model. In these moderation analyses, the respective stressor was included as independent variable, group (MD/TD) as dependent variable, and the protective factors “Social Support” and “Positive Family Climate”, respectively, as moderators.

## Results

### Group differences in stress domains and individual stressors

Results of the *χ*^*2*^-tests for the stress domains and individual stressors can be found in Table 2.

**Table 2:** Descriptive and statistical data for the stress domains and individual stressors in youths with MD and TD youths

Stress domains/individual stressors	Youths with MD (*n* = 100)	TD youths (*n* = 101)				
	%	%	*p*^a^	*χ*^2^	OR	95% CI for the OR
***Changes at home or at school***	71.4	45.5	< *.001*	13.71	3.00	[1.66, 5.38]
Repetition of a grade	35.7	12.9	< *.001*	14.18	3.76	[1.84, 7.68]
Had to change school	38.1	9.9	< *.001*	21.80	5.61	[2.60, 12.13]
Separation of parents	47.5	30.7	*.015*	5.92	2.04	[1.14, 3.64]
***Death of a loved one***^***b***^	14.0	5.9	.056	3.64	2.58	[0.95, 7.01]
Death of mother	3.0	0.0	n.a.^b^	3.08	7.29	[0.37, 142.93]
Death of father	5.0	3.0	n.a.^b^	0.54	1.72	[0.40, 7.40]
Death of a close friend	6.0	3.0	n.a.^b^	1.08	2.09	[0.51, 8.58]
***Experiences of violence***	51.5	20.4	< *.001*	20.67	4.14	[2.20, 7.78]
Beaten up at home	21.0	5.9	*.002*	9.80	4.21	[1.62, 10.94]
Yelled at and insulted at home	45.0	16.8	< *.001*	19.69	4.04	[2.10, 7.77]
Victim of violence	11.1	1.0	*.003*	8.98	12.38	[1.57, 97.80]
Victim of unwanted sexual acts	9.8	3.0	.055	3.69	3.47	[0.91, 13.24]
***Delinquent behavior***	21.2	9.0	*.016*	5.80	2.72	[1.18, 6.29]
Exercise of violence	12.1	3.0	*.015*	5.94	4.46	[1.22, 16.33]
In conflict with the police	14.0	5.9	.056	3.64	2.58	[0.95, 7.01]
***Psychological burdens during/after pregnancy***	48.8	30.6	*.013*	6.10	2.16	[1.17, 3.40]
Maternal postpartum depression	11.1	5.1	.136	2.22	2.33	[0.75, 7.24]
Maternal emotional distress during pregnancy	43.8	27.6	*.024*	5.09	2.05	[1.09, 3.82]
***Presence of an affective disorder in a first-degree relative***	47.5	7.9	< *.001*	39.23	10.51	[4.61, 23.93]
At least one affected parent	42.0	6.9	< *.001*	33.52	9.72	[4.10, 23.08]
At least one affected sibling	10.8	1.2	*.010*	6.68	9.94	[1.21, 81.49]
***Sociodemography***	77.0	58.8	*.008*	6.25	2.35	[1.24, 4.50]
Lower secondary education of the participant	18.0	5.0	*.004*	8.44	4.21	[1.50, 11.85]
Migrant background of the mother	11.6	12.1	.918	0.01	0.95	[0.39, 2.33]
Migrant background of the father	20.5	12.2	.132	2.27	1.85	[0.82, 4.13]
Unemployment of the mother	4.8	2.0	.298	1.08	2.43	[0.43, 13.62]
Unemployment of the father	2.6	2.2	.848	0.04	1.21	[0.17, 8.82]
Low academic qualification of the father	40.0	31.3	.219	1.51	1.47	[0.80, 2.70]
Low academic qualification of the mother	55.3	37.0	.013^c^	6.20	2.11	[1.17, 3.80]

*MD* major depression, *TD* typically developing, *OR* odds ratio, *CI* confidence interval

^a^Original *p*-values are reported. ^b^As the *χ*^*2*^-tests for the stress domain “Death of a loved one” was non-significant, follow-up *χ*^*2*^-tests for the individual stressors in this domain were not conducted. ^c^All significant *p*-values withstand Bonferroni-Holm correction except the stressor “Low academic qualification of the mother”. The stress domains are bolditalic. Significant *p*-values are italicized

### Identification of the most relevant stressors to predict case–control status

Table 3 displays the results of the binary logistic regression analysis. Note that the item “Presence of an affective disorder in a sibling” was not included in the binary logistic regression analysis, as not all statistical assumptions were met regarding the *χ*^2^-test. Likewise, the stressor “Low academic qualification of the mother” was not included because this factor did not withstand correction for multiple testing (see Table 2).

**Table 3:** Results of the binary logistic regression analysis

Predictors	*B*	*SE B*	*Wald Chi square*	*p*	OR	95% CI for the OR
Repetition of a grade	0.96	0.52	3.44	.064	2.60	[0.95, 7.14]
Had to change school	1.19	0.55	4.68	*.030*	3.28	[1.12, 9.59]
Separation of parents	0.10	0.44	0.05	.827	1.10	[0.47, 2.58]
Beaten up at home	0.50	0.74	0.47	.495	1.66	[0.39, 7.04]
Yelled at and insulted at home	0.58	0.51	1.29	.256	1.79	[0.66, 4.87]
Victim of violence	0.71	1.20	0.35	.553	2.04	[0.19, 21.46]
Exercise of violence	1.86	0.82	5.15	*.023*	6.41	[1.29, 31.88]
Maternal emotional distress during pregnancy	0.28	0.41	0.44	.505	1.32	[0.59, 2.97]
Presence of an affective disorder in at least one parent	2.53	0.51	24.46	< *.001*	12.60	[4.62, 34.40]
Lower secondary education of the participant	1.22	0.65	3.55	.060	3.38	[0.95, 12.03]

Hosmer and Lemeshow goodness-of-fit *χ*^2^-test indicated a good fit of the model. Significant *p*-values are italicized

*SE* standard error, *OR* odds ratio, *CI* confidence interval

A test of the full model against an intercept only model was statistically significant (*χ*^*2*^(10) = 71.34, *p* < .001). Together, the stressors predicted case–control status (MD/TD group). The model explained 44.8% (Negelkerke’s *R*^*2*^ = .448) of the variance in case–control status. Three variables emerged as significant and are the most relevant psychosocial stressors for predicting case–control status in the present sample: “Presence of an affective disorder in at least one parent”, “Exercise of violence”, and “Had to change school” (all *p*s < .05). The remaining variables did not emerge as significant in the analysis (all *p*s > .05). Based on the binary logistic regression model, 80.4% of youths with MD, 70.5% of the TD youths, and 76.0% of the participants overall were correctly classified.

### Group differences in protective factors

Results of the MANOVA for the three family climate scales (“Positive Family Climate”, “Activities”, and “Control”) and the *t*-test for the scale “Social Support” for MD and TD youth can be found in Table 4.

**Table 4:** Results for social support and the family climate scales in youths with MD and TD youths

Scale	Youths with MD (*n* = 100)	TD youths (*n* = 101)		
	*M (SD)*	*M (SD)*	*p*^d^	Effect size
Social support^a^	25.41 (*8.07*)	35.40 (*4.45*)	< *.001*	*d* = 1.53
Positive family climate^b^	28.68 (*9.65*)	39.78 (*5.88*)	< *.001*	*η*_*p*_^*2*^ = .33
Activities^c^	6.11 (*2.94*)	9.16 (*2.69*)	< *.001*	*η*_*p*_^*2*^ = .23
Control^c^	7.23 (*2.70*)	7.63 (*2.88*)	.292	*η*_*p*_^*2*^ = *.01*

*MD* major depression, *TD* typically developing, *M* mean, *SD* standard deviation

^a^Possible range: 0–40; ^b^Possible range: 0–52; ^c^Possible range: 0–16; ^d^Original *p*-values are reported. All significant *p*-values withstand Bonferroni-Holm correction. Significant *p*-values are italicized

The MANOVA including all three family climate scales revealed a significant effect of group (Pillai’s V = 0.35, *F*(3, 189) = 34.24, *p’* < .001, *η*_*p*_^*2*^ = .352). The follow-up ANOVAs revealed significant group differences for “Positive Family Climate” (*F*(1, 192) = 94.67), and “Activities” (*F*(1, 197) = 58.24). TD youths reported a higher positive family climate and more activities than depressed youths. The ANOVA on the effect of group on “Control” was non-significant (*F*(1, 197) = 1.12). Moreover, TD youths reported significant higher social support as depressed youths (*t*(194) = 10.73).

### Moderating effect of protective factors on the influence of specific stressors on MD

None of the moderation analyses with the independent variables “Had to change school”, “Exercise of violence”, and “Presence of an affective disorder in at least one parent”, respectively, the moderators “Social Support”, and “Positive Family Climate”, respectively, and group (MD/TD) as dependent variable emerged as significant (all *p*s for the interaction between the specific stressors and social support/positive family climate > .05).

## Discussion

The first aim of the present study was to investigate specific psychosocial stressors implicated in first-onset youth MD and to identify the most relevant stressors in this young patient group. The second aim was to examine whether social support and a positive family climate act as protective factors in youths with MD and moderate the relationship between specific psychosocial stressors and the disorder. In sum and in line with our hypothesis, we found that the proportion of youths who experienced various psychosocial stressors was higher in the MD compared with the TD group. The stressors “Presence of an affective disorder in at least one parent”, “Exercise of violence”, and “Had to change school” best predicted case–control status. We found that TD individuals experienced more social support and a more positive family climate than MD youths. However, no buffering effect of these protective factors on the relationship between the three above-mentioned particularly relevant stressors and MD was found.

### Occurrence of psychosocial stressors

Youths suffering from MD and TD youths were found to differ in a number of specific psychosocial stressors. With regard to the stress domain “Changes at home or at school”, it was shown that the proportion of youths who indicated that their parents were separated was higher in the MD than in the TD group. Results from the literature regarding parental separation are inconclusive, i.e., not all studies could identify parental separation, divorce, or living in a one-parent family as a risk factor for developing MD or depressive symptoms in youth [10–16]. It is important to note that possible intervening factors, such as secondary stressors, e.g., changes in the socioeconomic status, family conflict, as well as the loss of contact with one parent, may impact on the relationship between parental separation and youth MD [11, 42, 70].

The current study also revealed that within the stress domain “Changes at home or at school”, the repetition of a grade and change of school were more frequent in youths suffering from MD than in TD youths. Notably, having to change school emerged as one of the most relevant psychosocial stressors in the current sample predicting case–control status. Previous studies investigating specific school-related factors, such as repetition of a grade, yielded mixed results [16, 18]. The repetition of a grade or the change of school is often a result of low academic achievement and is discussed as risk factor for MD in youths and young adults, although this relationship seems to be bidirectional and particularly holds true for females [19–21]. In this context, we also found that more youths with MD than TD youths attend a school type in the lower secondary education system. Attending a school type in the lower secondary education system might—among other (socio)demographic factors—be drawn back to low achievement at school. Together, the findings indicate that school-related factors seem to be strongly implicated in youth MD. This highlights the importance of considering these factors in treatment and prevention approaches [71].

As expected and in line with the literature [22–24], experiences of violence, and specifically having been beaten up at home, having been yelled at and insulted at home, and having been a victim of violence, were more frequent in youths with MD as compared with TD individuals. In the current study, there was only a trend towards more youths with MD reporting having experienced unwanted sexual acts than TD controls, while prior studies have robustly identified sexual violence as a risk factor for youth MD [22, 24]. Our non-significant findings regarding this stressor might be due to the relatively low occurrence of unwanted sexual acts reported in the current study. It has been suggested that the experience of violence, especially early in life, may lead to neurobiological changes, e.g., as reflected in a dysregulation of the hypothalamic–pituitary–adrenal (HPA)-axis. This may predispose individuals to psychopathology, including a heightened vulnerability for the occurrence and maintenance of MD [72].

In the present study, delinquent behavior was more common in the MD than in the TD group, with more MD youths reporting being violent themselves than TD youths. Moreover, this factor was identified as one of the most important stressors for MD in youth. It has been suggested that the relationship between delinquent acts and depressive symptoms is bidirectional [26, 27]. In future studies, it would be worthwhile to evaluate possible mechanisms linking delinquent acts and youth MD. For instance, following the “failure model” it has been proposed that experiences of failure might mediate the relationship between delinquency and MD in youth. According to this notion, aggressive behavior or conduct problems can lead to experiences of failure (such as being rejected by peers or low achievement in school), which, in turn, might predispose youth to depressive symptoms or MD [73].

As hypothesized and in line with most earlier findings in youths with MD [28–30], we found that psychological burdens of the mother during pregnancy were more frequently reported in the MD group. Specifically, more mothers of youths with MD than mothers of TD controls reported emotional distress during pregnancy. One explanation is that emotional stress of the mother during pregnancy activates the maternal HPA-axis, which has been shown to influence the HPA-axis of the fetus, predisposing the offspring to MD [30]. Of note, unlike a number of previous studies [32–34], we did not find a relationship between postpartum depression of the mother and MD in the offspring. Given that we aimed to examine multiple stressors and protective factors and their relative association strength with youth MD, it was beyond the scope of the current study to conduct an interview or to apply separate self-rating scales for postpartum depression [74]. This approach would likely be more sensitive to detect this stressor than the dichotomous answer format applied in the current study. Apart from this issue, it has been shown that maternal postpartum depression and later maternal MD are related, leading to the suggestion that not postpartum depression per se but rather the subsequent depressive episodes or the genetic risk conveyed by having a parent with MD may predispose the offspring to the disorder [33]. In this context and in line with the literature [35–38], the present study found that the proportion of individuals with one or both parents or at least one sibling affected by MD was substantially higher in the MD compared with the TD group. Moreover, a parental history of an affective disorder emerged as the most important stressor for youth MD in the present study. Importantly, having a first-degree relative with an affective disorder acts both as a genetic and environmental risk factor, with approximately 40% of the variance in female MD during youth being explained by genetic factors, whereas unique environmental factors seem to contribute with approximately 60% [75].

### Protective factors

Results from the current study indicate that TD compared with MD youths experience more social support and a more positive family climate. These factors have been previously discussed as protective factors regarding development of youth MD and depressive symptoms [13, 18, 45, 49, 76]. In the present study, we did not find evidence that social support and a positive family climate have a buffering effect on the relationship between specific stressors that best predicted case–control status (“Presence of an affective disorder in at least one parent”, “Exercise of violence” and “Had to change school”) and MD. Our results contradict the general assumption of a buffering effect of social support on the relationship between stressors and MD [46]. However, our findings are in line with a number of other studies that also did not find a buffering effect of social support, suggesting that stressors and protective factors exert independent effects on depressive symptoms and MD in youth [47–49]. In future studies that examine potential buffering effects of protective factors in youth with a clinical diagnosis of MD, it would be worthwhile to consider neurobiological in addition to psychosocial stressors to account for the multi-faceted etiology of the disorder.

### Limitations and strengths

The results of the present study need to be considered in light of some limitations. First, as we assessed stressors in a cross-sectional design, we cannot make inferences regarding the directionality of the relationship between stressors and depression status. In line, it is conceivable that some of the stressors assessed might have occurred during the depressive episode. However, to reduce this possibility, we only included patients with a current first-onset depressive episode. Second, psychosocial stressors were assessed in part based on self-report. It is likely that being in a state of depressed mood might lead to cognitive biases, such as memory/recall bias, making MD patients prone to remember or report more negative events which are congruent with their current negative mood [77]. However, while this limitation is inherent to measures of retrospective self-report, we assume that a potential recall bias in our sample of depressed youths would be smaller than in previous studies predominantly examining depressed adults due to the shorter time period between occurrence of the stressor and the assessment thereof.

Despite these limitations, the current study substantially adds to previous studies in the field by investigating youths with a clinical diagnosis of MD and by examining a wide range of individual psychosocial stressors in conjunction with protective factors. A particular strength of the study is that we included a very well-characterized clinical sample of youths who were all currently treated for a first episode of MD. A further strength of the study is that we not only collected self-report data, but also assessed information reported by the parents, including birth related-factors and a family history of MD.

## Conclusions

Extending previous studies in non-clinical adolescent analogue or adult MD samples, we found that a number of psychosocial stressors more commonly occur in youths with MD and substantially explain variance in case–control status. These results indicate that psychosocial stressors play an important role in this young patient group. In particular, it was shown that school-related factors, violence, affective disorders in the family, as well as sociodemographic factors are related to MD in youth. Identification of relevant and frequently occurring stressors in the context of youth MD is highly important as these factors can represent specific targets in prevention and treatment efforts. For example, one promising approach would be to train high-risk youths (e.g., with a family history of MD) in adequately coping with distinct (e.g. school-related) stressors. Future longitudinal studies should investigate the causal order of psychosocial stressors in relation to MD during youth and examine additional (e.g., neurobiological) aspects influencing the relationship between psychosocial stressors and youth MD.

## Supplementary information

**Additional file 1.** Frequency of current and past comorbid diagnoses in the MD group.

**Additional file 2.** Psychometric properties of the “Family climate scale” and the “Social support scale”.

**Additional file 3.** Data analysis.

## Data Availability

Data in our study contain sensitive patient information, such as sociodemographic information and comorbidities. Since patients could possibly be identified by making our raw data publicly available, ethical principles of protecting patient confidentiality would be breached. Therefore, raw data cannot be made publicly available. Relevant data and information, such as methods and materials used, as well as description of the sample, can be found in the article as well as in the additional material. Additional materials and aggregated data can, however, be made available upon request (contact: Charlotte.Piechaczek@med.uni-muenchen.de).

## Abbreviations

MD: Major depression
TD: Typically developing
SLE: Stressful life event
ADHD: Attention deficit hyperactivity disorder
DIKJ: Depression Inventory for Children and Adolescents
BDI: Beck Depression Inventory
MEL: Munich event list
MANOVA: Multivariate analysis of variance
ANOVA: Analysis of variance
OR: Odds ratio
CI: Confidence interval
SE: Standard error
M: Mean
SD: Standard deviation
HPA-axis: Hypothalamic-pituitary-adrenal axis
