# Supplementary material for: Psychosocial stressors and protective factors for major depression in youth: evidence from a case–control study
Source: Child Adolesc Psychiatry Ment Health. 2020 Feb 8;14:6. doi: 10.1186/s13034-020-0312-1 (PMC7007652; doi:10.1186/s13034-020-0312-1)
Supplement: Supplementary file 1 — Additional file 1. Frequency of current and past comorbid diagnoses in the MD group. [file 13034_2020_312_MOESM1_ESM.pdf]

Additional file 1

Frequency of current and past comorbid diagnoses in the MD group

	Frequencies	
	Current diagnosis	Past diagnosis
Oppositional defiant disorder	7.5%	5.4%
Conduct disorder	2.2%	1.1%
Separation anxiety disorder	2.2%	4.3%
Specific anxiety disorder	22.1%	7.5%
Social anxiety disorder	16.0%	1.1%
Generalized anxiety disorder	4.3%	1.1%
Panic disorder	1.1%	1.1%
Agoraphobia	4.3%	1.1%
Posttraumatic stress disorder	0%	5.3%
Dysthymia	0%	9.7%
