# Supplementary material for: Psychosocial stressors and protective factors for major depression in youth: evidence from a case–control study
Source: Child Adolesc Psychiatry Ment Health. 2020 Feb 8;14:6. doi: 10.1186/s13034-020-0312-1 (PMC7007652; doi:10.1186/s13034-020-0312-1)
Supplement: Supplementary file 2 — Additional file 2. Psychometric properties of the “Family climate scale” and the “Social support scale”. [file 13034_2020_312_MOESM2_ESM.pdf]

Additional file 2

Psychometric properties of the "Family climate scale" and the "Social support scale"

The factor analysis for the social support scale revealed one factor (Kaiser-Meyer-Olkin, KMO = .94). The factor explained 63.23% of the variance. The factor analysis for the family climate scale revealed three factors (KMO = .92). The three factors together explained 52.61% of the variance. The items clustering on the first factor suggested that it represents "Positive family climate" (e.g., "In our family everyone has the feeling that one is listening to him and pays attention to him."), factor 2 represents "Activities" (e.g., "Our family goes about activities, like for example going to the movies, visit sports events or go on trips."), and factor 3 represents "Control" (e.g.,"At home it is determined very precisely what may be done and what may not be done.").

In a next step, the internal consistency of the scales "Social Support", "Positive family climate", "Activities", and "Control" was calculated based on the present sample. Internal consistency was excellent for "Social support" (Cronbach's α = .94), excellent for "Positive family climate" (Cronbach's α = .92), good for "Activities" (Cronbach's α = .80), and acceptable for "Control" (Cronbach's α = .70).
