# Supplementary material for: Psychosocial stressors and protective factors for major depression in youth: evidence from a case–control study
Source: Child Adolesc Psychiatry Ment Health. 2020 Feb 8;14:6. doi: 10.1186/s13034-020-0312-1 (PMC7007652; doi:10.1186/s13034-020-0312-1)
Supplement: Supplementary file 3 — Additional file 3. Data analysis. [file 13034_2020_312_MOESM3_ESM.pdf]

**Additional file 3**

## Data analysis

*Establishing stress domains*

Due to the substantial number of stressors included in the present investigation, we defined several psychosocial stress domains under which the specific psychosocial stressors were grouped. To subsume the individual stressors under the stress domains, we conducted an exploratory factor analysis and considered the content of the individual stressors. This step yielded the following stress domains: “Changes at home or at school”, “Experiences of loss”, “Experiences of violence”, “Delinquent behavior”, “Psychological burdens during/after pregnancy”, “Affective disorders in a first-degree relative”, and “Sociodemography”. The presence of a stress domain was established in an individual if at least one of the stressors subsumed under each stress domain was present (i.e., 0 vs. 1 scoring). We then investigated differences between MD and TD participants concerning the presence of these stress domains to allow data reduction and to avoid problems inherent to multiple testing.
